# Assessment of the skin tissue structure using an ultrasonic diagnostic imaging device in patients undergoing open surgery

**DOI:** 10.1111/srt.12727

**Published:** 2019-05-29

**Authors:** Fumiya Oohashi, Takashi Suto, Hiroki Matsui, Ken Shirabe, Yoko Uchida

**Affiliations:** ^1^ Department of Nursing, Graduate School of Health Sciences Gunma University Gunma Japan; ^2^ Department of Anesthesiology Gunma University Graduate School of Medicine Gunma Japan; ^3^ Department of Laboratory Sciences Gunma University Graduate School of Health Sciences Gunma Japan; ^4^ Department of General Surgical Science Hepato‐Biliary‐Pancreatic Surgery Gunma University Graduate School of Medicine Gunma Japan; ^5^ Present address: Department of Clinical Nursing, Graduate Course of Nursing Science, Division of Health Sciences, Graduate School of Medical Sciences Kanazawa University Kanazawa Japan

**Keywords:** cobblestone appearance, epidermal/dermal tissue, increased echogenicity, opaque layer structure, open surgery, subcutaneous tissue, ultrasonic diagnosis device

## Abstract

**Background:**

Perioperative skin injury is a major issue; therefore, several preventative measures have been developed. However, no previous studies have visualized the effects of stromal edema caused by surgical invasion of skin tissue, and therefore, the details remain unknown. We used an ultrasonic diagnostic imaging device to clarify changes in the skin tissue structure of patients after open surgery.

**Materials and Methods:**

Twenty subjects who underwent open hepatectomy were enrolled. We selected the lateral abdomen, upper arms, and lower legs as ultrasonic imaging measurement sites. We performed measurements on the day before surgery and on postoperative days 1, 3, and 5. We calculated the epidermal/dermal tissue thickness, subcutaneous tissue thickness, and skin tissue thickness. We performed a one‐way analysis of variance with repeated measurements for each of the postoperatively measured values on the basis of the preoperative values. Significantly different variables were subjected to the Bonferroni method. We evaluated ultrasonic imaging findings and skin injury.

**Results:**

Epidermal/dermal tissue thickness at all measurement sites exhibited sustained thickening on postoperative day 5 compared to that preoperatively. The lateral abdomen exhibited thickening of the subcutaneous tissue and skin tissue on postoperative day 1. In addition, increased echogenicity, increased opacity of the layer structure, and a cobblestone appearance occurred during the postoperative course. Postoperatively, 80% of subjects exhibited skin injury.

**Conclusion:**

We evaluated the effects of surgical invasion on skin tissue over time. Continual observation and protective skincare are necessary near the surgical wound, where significant invasiveness occurs. Prevention of skin injury due to skin tissue thickening requires further study.

## 1 INTRODUCTION

Skin tissue, which is composed of the epidermis, dermis, and subcutaneous tissue, protects the body from various stimuli and from encroachment by water, microorganisms, and foreign substances. In addition, it is the largest organ of the body, with the epidermis and dermis alone comprising approximately 14% of body weight.^1^, ^2^

In recent years, skin tissue injuries such as medical device‐related pressure ulcers,^3^ bed sores,^4^ and skin tears^5^ have been noted as major issues. As a result of a variety of measures that were implemented, the bedsore occurrence rate has decreased significantly in Japan; however, it has been found that it has not decreased in the setting of perioperative care.^6^

One characteristic of the perioperative period is that the effects of various forms of surgical stress result in the pooling of interstitial fluid postoperatively. A cause of this is believed to be the localized inflammation and exudate accumulation caused by tissue injury.^7^ In addition, rupture of the vascular endothelial cells and surface layer of capillaries increases vascular permeability, resulting in the movement of moisture from the vascular lumen to the stroma, causing stromal edema.^8^ Furthermore, stress hormones are activated by pain during surgery. Adrenocorticotropic hormone, cortisol, antidiuretic hormone, renin‐angiotensin‐aldosterone, epinephrine, and others have a common role in body sodium (Na) and moisture retention; together with intraoperative infusions, they exacerbate stromal edema.^9^ The amount of moisture movement into the interstitial fluid is greatest during upper abdominal surgery, such as open hepatectomy, which was the subject of this study in particular,^10^ and increased bleeding results in larger amounts of fluid and blood transfusion, further leading to a collapse of the balance of body fluid circulation.

No previous studies have elucidated the effects of stromal edema caused by these stresses on skin tissue; therefore, we considered it necessary to visualize the changes that occur through postoperative progression. We focused on evaluating the skin tissue structure using an ultrasonic diagnostic imaging device. Such devices are widely used in the clinical field to observe internal organs. They are effectively utilized for evaluating muscle mass in the legs,^11^ lymphedema,^12^ and even surface tissues and for diagnosing pressure ulcers.^13^ The technique is noninvasive and allows real‐time evaluation, and its accuracy and utility have been proven.

## 2 MATERIALS AND METHODS

### 2.1 Definition of terms

In this study, not only changes in epidermal/dermal tissue thickness, subcutaneous tissue thickness, and skin tissue thickness before and after surgery but also in ultrasonic imaging findings of the skin tissue structure were evaluated.

### 2.2 Study design

This was a prospective observational study conducted at the hospital affiliated with the medical department at Gunma University, which cooperated with and consented to participation in this study. The survey period was from June through October 2017.

### 2.3 Subjects

Subjects were 20 patients who planned to undergo open hepatectomy with a planned operative time of at least 8 hours and whose primary physicians provided consent to cooperate with the study.

### 2.4 Medical record survey

#### 2.4.1 Basic subject characteristics

The basic characteristics obtained from medical records were age, sex, body mass index, primary condition, infection type, nutritional state (albumin and total protein), respiratory function status (vital capacity as a percent of predicted [%VF] and forced expiratory volume in 1 second as a percent of forced vital capacity [FEV_1.0%_]), smoking history, medical history, and degree of liver damage.

#### 2.4.2 Surgical data

We collected the surgical data and water intake/output balance data from the medical records, anesthesia charts, surgical nurse records, and intensive care unit records.

### 2.5 Methodology for ultrasonic imaging measurements

#### 2.5.1 Measurement times

A total of four measurements were obtained. Measurements were performed using the ultrasonic diagnostic device the day before surgery, postoperative day 1, postoperative day 3, and postoperative day 5 (the day of the surgery was defined as postoperative day 0).

#### 2.5.2 Ultrasonic diagnostic device

Ultrasonic tomographic imaging was performed using the S Series S‐Nerve Ultrasound System SonoSite for all subjects. The ultrasonic probe was a 15 to 16 MHz linear model with superior visualization of surface tissues. A gel pad (2 × 9 cm; Aquaflex ultrasound gel pad; Parker Laboratories, Inc) was used at the measurement site; this gel pad enabled clear visualization of the most superficial layer of the skin, avoidance of excessive pressure on the skin tissue during measurements, and prevention of changes in skin thickness caused by the technique when the probe was manipulated. In addition, a mark was made using a medical marker (Surgical Skin Marker; Bonimed), with the consent of the subjects, so that the same site could be measured on all measurement days.

#### 2.5.3 Study protocol

To ensure the reliability and validity of the measured values without imposing an undue burden on the subjects while studying them, we prepared a protocol for the measurement technique/methodology on the basis of previous studies and the advice of experts, anesthesiologists, and clinical laboratory technicians. Then, the survey contents and techniques/methodologies were confirmed using the protocol for healthy subjects. We repeatedly verified that there would be no undue burden on subjects postoperatively while revising the protocol. The revised protocol was again examined by researchers and then adopted as the measurement procedure.

#### 2.5.4 Site and body position for measurement

There were three measurement sites: the lateral abdomen, upper arms, and lower legs. The basic body position comprised a horizontal head and the supine position. When measuring the lateral abdomen (10 cm proximal from the left anterior superior iliac spine), the subject was prompted to breathe deeply and relax. In such a state, the arms were held in the same position as that during the arm measurements. When measuring the arms (10 cm proximal, linking the midpoint of the ulna and radius and the midpoint of the medial epicondyle and the lateral epicondyle of the humerus), they were bent to 90 degrees, with the hands held at the chest and the measurement being performed at the posterior surface. When measuring the lower legs (10 cm distal, linking the midpoint of the patella and the third digit), the legs were bent to 90 degrees, and the measurement was performed at the posterior surface.

#### 2.5.5 Measurement methods

A gel pad was placed at each measurement site. An ultrasonic probe was in contact with each pad, and the longitudinal axis and perpendicularity were maintained. When contacting the ultrasonic probe, the pressure was that caused by its own weight for all subjects, thereby consistently avoiding excessive pressure on the skin tissue. The ultrasonic probe was grasped between the first and third digits of the dominant hand, and the fifth digit was lightly placed against the body surface to maintain stability during the measurements. The gain was set to auto gain, and the depth was set to 4.0 cm. Images were obtained in the horizontal direction; the imaging site was shifted in intervals of 5.0 mm to acquire six images per measurement point, and the resulting values were averaged. The person performing the measurement was instructed by an anesthesiologist and a clinical laboratory technician.

### 2.6 Ultrasonic image analysis

Ultrasound images were analyzed using ImageJ (National Institutes of Health, Bethesda, Maryland, USA) by the researcher and the research assistant who were instructed by an anesthesiologist and clinical laboratory technician. The method of distinguishing between the epidermal/dermal tissue layers and the subcutaneous tissue layer using image coloration was as follows: (a) image, type, 8 bit; (b) image, lookup table, 16 colors; and (c) image, adjust, threshold. The method of measuring the epidermal/dermal tissue thickness and subcutaneous tissue thickness was as follows: (a) if the depth is 4.0 cm, then measure 1.0 cm; (b) analyze, set scale; (c) known distance (select 1.0); (d) designate cm as the unit; (e) measure the epidermal/dermal tissue thickness and subcutaneous tissue thickness; (f) analyze and measure; and (g) measure three locations each at the left, right, and center of the epidermal/dermal tissue thickness and subcutaneous tissue thickness and calculate the mean. The method of measuring skin tissue thickness was as follows: the values of the combination of the average values of the epidermal/dermal tissue thickness and subcutaneous tissue thickness for each measurement day were calculated. All of these methods were performed using Image J (National Institutes of Health).

### 2.7 Ultrasonic imaging findings

We evaluated the presence of opaque layer structures, increased echogenicity of subcutaneous tissue, ruptured fascia, and the cobblestone appearance of the ultrasound image. The researcher and research assistant performed separate evaluations, and the findings used were those on which both parties agreed. The presence and state of the skin injury were evaluated according to the surgical nurse records, anesthesia charts, medical records, and observations during measurements.

### 2.8 Data analysis

The epidermal/dermal tissue thickness, subcutaneous tissue thickness, and skin tissue thickness values were subjected to the Shapiro‐Wilk test to confirm their normality. A comparison of changes in the epidermal/dermal tissue thickness, subcutaneous tissue thickness, and skin tissue thickness over time was performed using one‐way analysis of variance (ANOVA) with repeated measures for the values from each postoperative measurement day on the basis of the preoperative values. The factors for which a significant difference was found were subjected to multiple comparisons (Bonferroni method). The significance threshold was less than 5%. Statistical processing was performed using IBM SPSS Statistics version 23.

Oral and written explanations were provided to the subjects and their families regarding the study purpose and methods, protection of personal information, free cooperation in the study, maintenance of anonymity, and publication of study results. We obtained written signatures as verification of consent from the subjects. In addition, we immediately halted the study procedures if cooperation in the survey could not be obtained, such as if the subject reported pain or nausea during the measurement. The study was performed with the approval of the institutional review board of Gunma University Hospital (approval number 1536).

## 3 RESULTS

Basic characteristics of the 20 subjects are shown in Table 1. Based on the average age of the subjects, elderly people were targeted. The liver reserve capacity was maintained in relatively good condition.

**Table 1:** Patient characteristics

Patient characteristics (n = 20)	Mean ± SD	N (%)	Min	Max	Median
Age, years	65.9 ± 9.6		46.0	83	67.5
Sex, male		16 (80%)			
Sex, female		4 (20%)			
BMI, kg/m^2^	23.8 ± 2.0		20.2	27.7	23.6
Hepatocellular carcinoma		5 (25%)			
Metastatic hepatic cancer		5 (25%)			
Hilar cholangiocarcinoma		4 (20%)			
Gallbladder carcinoma		3 (15%)			
Hepatocellular carcinoma and intrahepatic cholangiocarcinoma		2 (10%)			
Intrahepatic cholangiocarcinoma		1 (5%)			
Child‐Pugh classification A		20 (100%)			
Degree of liver damage, classification A		18 (90%)			
Degree of liver damage, classification B		2 (10%)			
Albumin, g/dL	3.8 ± 0.3		2.8	4.6	3.9
Total protein, g/dL	6.7 ± 0.5		5.1	7.5	6.8
%VC	106.3 ± 28.5		84.7	137.8	110.3
FEV1.0%	74.8 ± 6.8		65.2	85.9	75.9

BMI, body mass index; %VC, vital capacity as a percent of the predicted vital capacity; FEV1.0%, forced expiratory volume in 1 s as a percent of forced vital capacity.

**Figure 1:**
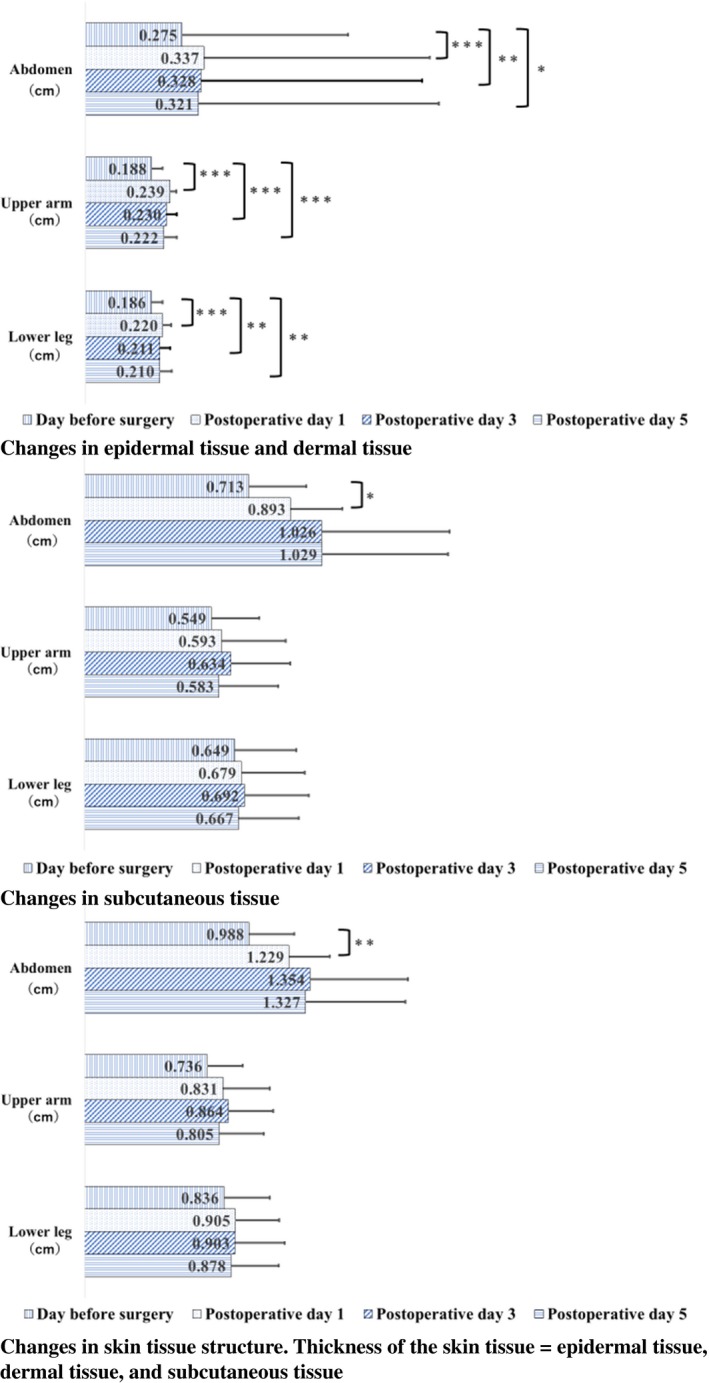
Changes in skin thickness measurements using ultrasound imaging (n = 19). Repeated measures ANOVA and Bonferroni correction: *P<0.05; **P<0.01; ***P<0.001 [Colour figure can be viewed at http://wileyonlinelibrary.com]

Surgical data are provided in Table 2. The operative time was more than 7 hours, and the anesthesia time was approximately 9 hours. In addition, the average postoperative water balance was more than 2755 mL (standard deviation, ±797.8 mL), which was excessive. Confirmation of normality using the Shapiro‐Wilk test is shown in Table 3. Comparisons of skin tissue throughout postoperative progression at each measurement site are described in Figure 1. Epidermal/dermal tissue thickness at all measurement sites exhibited sustained thickening on postoperative day 5 compared to that preoperatively. Thickening of the subcutaneous tissue and skin tissue in the lateral abdomen was observed on postoperative day 1.

**Table 2:** Surgical data

Postoperative course (n = 20)	Mean ± SD	Min	Max	Median
Operative time, min	437.9 ± 125.6	261.0	659.0	386.5
Anesthesia time, min	538.9 ± 125.8	356.0	729.0	495.5
Resection liver weight, g	456.4 ± 386.0	72.0	1447.0	337.0
Liver ligament ischemic time, min	94.0 ± 53.2	43.3	251.6	80.9
Amount of bleeding, g	297.0 ± 436.2	38.0	2002.0	436.2
Amount of urine, mL	571.1 ± 327.5	100.0	1150.0	565.5
Intraoperative infusion balance, mL	2775.3 ± 797.8	1625.0	4765.0	2620.5
Length of stay (intensive care unit), days	3.4 ± 1.3	2.0	7.0	3.0
Length of stay (postoperative), days	18.3 ± 18.9	8.0	88.0	11.0
Length of stay (hospital), days	28.4 ± 27.8	10.0	109.0	15.0

**Table 3:** Shapiro‐Wilk test results (*F* = 12.000)

Measuring date	Skin tissue structure classification	Ultrasound image measurement site					
		Abdomen		Upper arm		Lower leg	
		Statistics	*P*‐value	Statistics	*P*‐value	Statistics	*P*‐value
The day before surgery	a	0.965	0.853	0.904	0.180	0.871	0.067
	b	0.915	0.246	0.930	0.375	0.852	0.039
	c	0.918	0.269	0.968	0.891	0.833	0.023
Postoperative day 1	a	0.805	0.011	0.861	0.051	0.907	0.194
	b	0.956	0.724	0.903	0.175	0.969	0.897
	c	0.930	0.380	0.912	0.226	0.936	0.445
Postoperative day 3	a	0.856	0.044	0.892	0.123	0.913	0.232
	b	0.777	0.005	0.949	0.628	0.920	0.288
	c	0.782	0.006	0.921	0.293	0.914	0.237
Postoperative day 5	a	0.955	0.708	0.953	0.688	0.938	0.473
	b	0.800	0.009	0.922	0.300	0.899	0.155
	c	0.789	0.007	0.950	0.640	0.925	0.329

a, epidermal tissue and dermal tissue; b, subcutaneous tissue; c, skin tissue structure.

**Figure 2:**
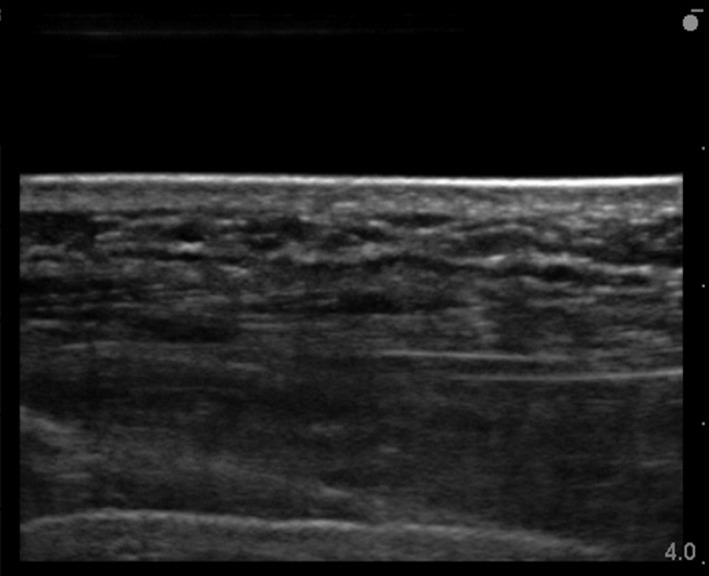
Identification of the normal skin tissue structure the day before surgery

**Figure 3:**
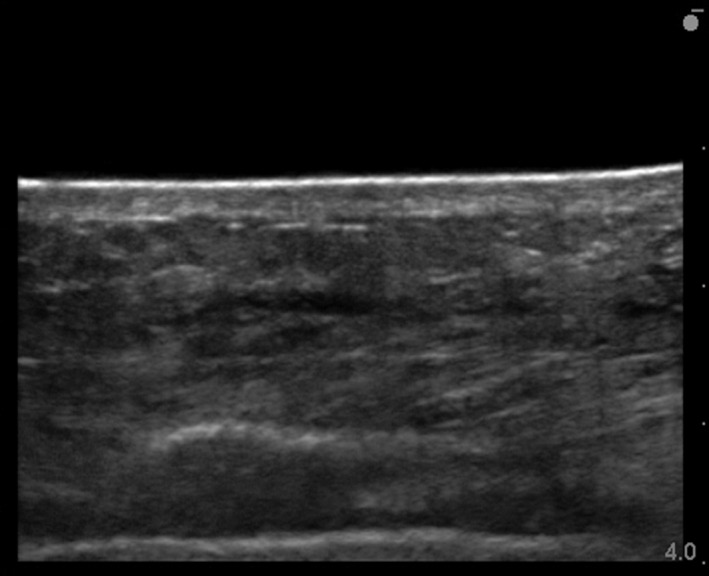
Loss of superficial fascia. Postoperative day 1

**Figure 4:**
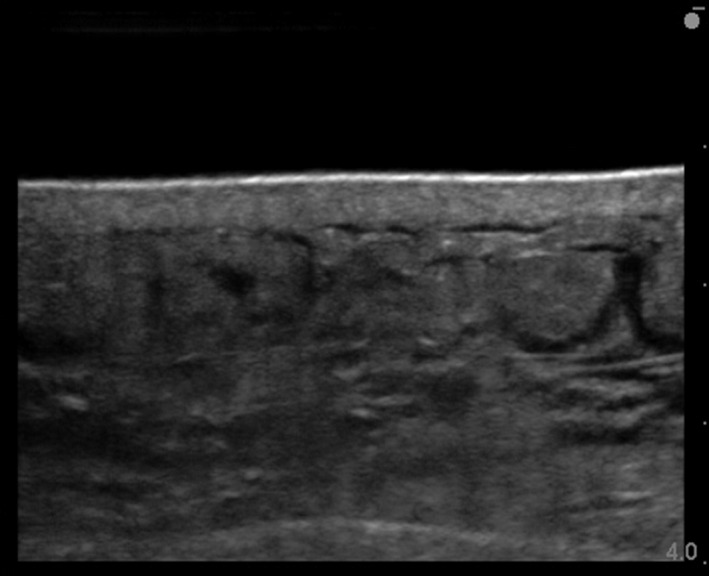
Cobblestone appearance. Postoperative day 3

**Figure 5:**
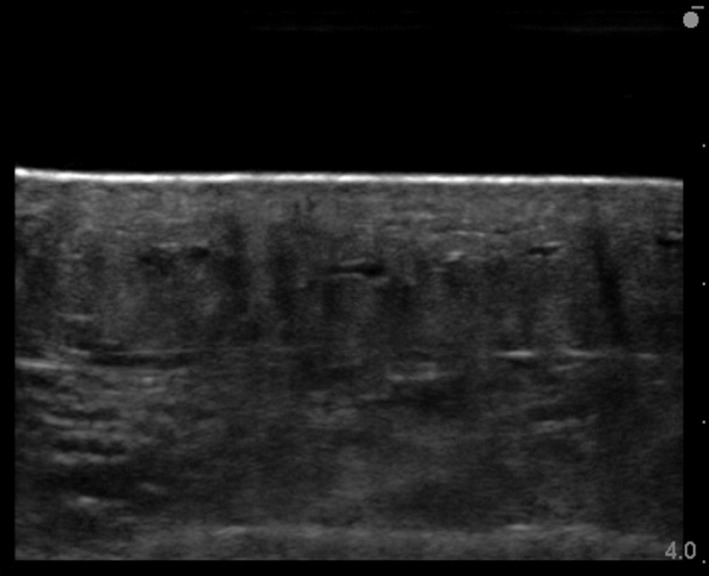
Opaque layer structure. Postoperative day 5

The lateral abdomen exhibited a higher frequency of increased echogenicity of subcutaneous tissue, cobblestone appearance, and increased opacity of the layer structure compared with the other measurement sites (Table 4). Furthermore, the transition of the abdominal echo image of the same patient is shown (Figures 2, 3, 4, 5). Skin injury occurred in 16 of the 20 subjects (80%) (Table 5). Of the types of skin injury, the most common was epidermolysis bullosa, which was found in 11 subjects (69%), followed by blistering, which was found in 2 subjects (12%), internal bleeding, which was found in 2 subjects (12%), and redness, which was found in 1 subject (7%). The skin injury occurred most commonly in nine locations of the abdomen and five locations of the thorax where the surgical drapes were adhered, followed by two locations on the thigh, one location on the back, one location on the neck, and one location on the upper arm. Skin injury occurred most commonly immediately after surgery (8 cases; 50%), followed by day 3 postoperatively (4 cases; 25%), day 5 postoperatively (3 cases; 19%), and day 2 postoperatively (1 case; 6%).

**Table 4:** Ultrasound imaging findings

Measurement day	Ultrasound imaging findings	Ultrasound image measurement site N (%)		
		Abdomen	Upper arm	Lower leg
Day before surgery (n = 20)	d	1 (5%)	0 (0%)	0 (0%)
	e	2 (10%)	1 (5%)	2 (10%)
	f	0 (0%)	0 (0%)	0 (0%)
	g	0 (0%)	0 (0%)	0 (0%)
Postoperative day 1 (n = 20)	d	6 (30%)	1 (5%)	0 (0%)
	e	11 (55%)	3 (15%)	3 (15%)
	f	0 (0%)	1 (5%)	0 (0%)
	g	1 (5%)	0 (0%)	0 (0%)
Postoperative day 3 (n = 20)	d	8 (40%)	1 (5%)	0 (0%)
	e	11 (55%)	1 ((5%)	2 (10%)
	f	1 (5%)	0 (0%)	1 (5%)
	g	3 (15%)	0 (0%)	0 (0%)
Postoperative day 5 (n = 19)	d	9 (45%)	1 (5%)	1 (5%)
	e	11 (55%)	2 (10%)	3 (15%)
	f	2 (10%)	0 (0%)	1 (5%)
	g	3 (15%)	0 (0%)	0 (0%)

d, opaque layer structure; e, high brightness of the subcutaneous tissue; f, rupture of the muscle membrane; g: cobblestone.

**Table 5:** Postoperative skin injury (n = 16)

Skin injury date		Skin injury occurrence site		Type of skin injury	
Immediately after surgery	8 (50%)	Abdomen	9 (45%)	Epidermolysis	11 (69%)
Postoperative day 3	4 (25%)	Chest	5 (25%)	Skin blisters	2 (12%)
Postoperative day 5	3 (19%)	Femur	2 (10%)	Skin bleeding	2 (12%)
Postoperative day 2	1 (6%)	Other sites	4 (20%)	Skin redness	1 (7%)

## 4 DISCUSSION

Changes in the skin structure of patients who underwent highly invasive open surgery led to the discovery of defining characteristics. As a result of using an ultrasonic imaging diagnosis device to compare the skin tissue thickness at the lateral abdomen, upper arm, and lower leg, we found that the epidermal/dermal tissue thickened at all measurement sites. Interstitial fluid was present between cells and was considered to exist in marked quantities in dermal tissue located underneath the epidermal tissue. Therefore, we were able to confirm postoperative stromal edema in the epidermal/dermal tissue. One cause of thickening was believed to be the accumulation of exudates from the hepatic resection surface. Exudates flow from sites of organ excision, such as the hepatic resection surface. Exudates primarily result from localized vascular permeability caused by the inflammatory response, and it has been shown that tissue edema occurs at surgical sites.^14^ Therefore, we observed marked thickening of each layer of skin tissue at the lateral abdomen, which was near the surgical wound.

A third space, which refers to moisture that forms tissue/organ edema via nonfunctional extracellular fluid, formed as a result of increased vascular permeability. With intracellular fluid as the first space and extracellular fluid as the second space, the increased vascular permeability due to surgical invasion and the resulting movement of intravascular moisture out of the vasculature resulted in edema in organs adjacent to the surgical site and those further away.^15^ It is possible that edema was most prominent at the lateral abdomen because the amount of moisture transfer to the third space was highest at sites of highly invasive surgery. Furthermore, Tatara et al used a simulation model to study edema in relation to the amount of fluid infusion over time.^16^ Their results indicated that although edema did not occur during brief and rapid fluid infusion, the longer the period of infusion, the more pronounced the edema became. Furthermore, it was shown that for surgeries exceeding 8 hours, edema could not be avoided, even if intraoperative fluid infusions were restricted and the fluid was retained in the stroma.^10^ Therefore, an increased total amount of fluid infusion due to long‐term anesthesia may also affect skin tissue thickening. Moreover, postoperative sedentariness and a recumbent posture caused by pain are considered causes of epidermal/dermal tissue thickening at the upper arm and lower leg. Gravity acting on the body when sedentary and recumbent could cause interstitial fluid to move to and accumulate in the epidermal/dermal tissues located at the outermost layers of skin tissue.

Compared to the imaging findings at the upper arm and lower leg, the lateral abdomen exhibited more marked increases in echogenicity of the subcutaneous tissue and in opacity of the layer structure. In addition, a cobblestone appearance was only identified at the lateral abdomen. According to previous studies, increased echogenicity of subcutaneous tissue according to magnetic resonance imaging and ultrasonic imaging findings was a finding of interlobular fibrosis and accumulation of bodily fluids; however, these adipose lobules have also been found to contain almost no accumulation of body fluids.^16^ It has been reported that this reflects the involvement of vascular components owing to the breakdown of the capillary network, thus leading to the detection of increased brightness due to stronger acoustic backscattering.^17^

Increased opacity of the layer structure is considered to result from swelling of the subcutaneous tissue and the loss of superficial fascia due to accumulated body fluids.^18^ This has also been shown to occur because of body fluid accumulation in the epidermal and dermal tissues when edema causes particularly pronounced firmness.^13^ It has been shown that with a cobblestone appearance marked edema increases the attenuation of ultrasonic waves, making the deep muscle layers indistinct and making the distinction between the low‐echogenicity region of interstitial fluid and the high‐echogenicity region of subcutaneous fat more clearly visible.^19^ As described, previous studies have indicated that body fluid accumulation would increase^13^ and that the proteins in the retained fluids would lead to increased adipose tissue and formation of fibrous tissue in between.^20^, ^21^ Therefore, because such findings occurred to a greater extent at the lateral abdomen, which is the site of highly invasive surgery, we believe it is highly likely that these characteristic findings reflect the postoperative accumulation of interstitial fluid; however, the present study could not confirm this.

Stromal edema of the epidermal/dermal tissue layers alters the structure of the skin tissue, reduces elasticity, and leads to weakness.^22^ The results of this study indicated that a number of cases of postoperative epidermolysis were caused by physiological factors such as thickened skin tissue layers and skin moisture from perspiration, chemical factors caused by the application of medical tape for an extended duration, and physical stimuli, such as when the tape was peeled off the skin. An existing survey of skin tears also found that approximately 40% involved epidermolysis caused by medical tape as a result of thinning and edema of skin tissue.^23^ Furthermore, a study by Imazuru^24^ found that skin injury where drapes were adhered and at sites prone to pressure ulcers when lying supine occurred in 85% of patients. Therefore, it is important to improve surgical draping materials, introduce preoperative patch testing, and use agents to protect skin such as peeling adhesives to achieve perioperative management that does not cause skin injury.

This study succeeded in providing an understanding of the thickening of each individual skin tissue layer using an ultrasonic diagnostic imaging device. As a result, it is necessary to develop focused measures for preventing perioperative skin injury by studying skin injury prevention care for each layer and to elucidate the relationship between the characteristic stromal edema and skin injury. However, this study was performed using a limited number of patients at a single facility; therefore, it was not possible to control for the surgical procedure, amount of blood loss, and degree of surgical invasiveness, thereby making the results difficult to generalize.

## 5 CONCLUSION

We performed an objective evaluation of the skin tissue structure using an ultrasonic diagnostic imaging device to visualize thickening of the epidermal/dermal tissue layers at the lateral abdomen, upper arm, and lower leg as a result of surgical invasion. Furthermore, we identified subcutaneous tissue thickening and characteristic stromal edema at the lateral abdomen, which is the site of highly invasive surgery. As a result, we showed that skin tissue is weakened postoperatively, particularly at the site of the surgical wound, and is more easily damaged. In the future, more patients should be studied to determine the effects of differences in surgical procedures and methods of anesthetization on changes in the skin tissue structure on the basis of age and physical conditions.

## CONFLICT OF INTEREST

The present study was performed with a grant from the Yasuda Medical Foundation.
